# The correlation between perceived psychosocial stress and stroke: a meta-analysis

**DOI:** 10.3389/fneur.2025.1639076

**Published:** 2026-01-02

**Authors:** Yukai Wang, Xiaohua Shi, Lei Xu

**Affiliations:** Department of Neurology, China-Japan Union Hospital of Jilin University, Changchun, Jilin, China

**Keywords:** stress, stroke, ischemic, hemorrhagic, case–control study, prospective cohort study

## Abstract

**Objective:**

This meta-analysis aimed to evaluate the association between perceived psychosocial stress and stroke.

**Methods:**

We systematically searched PubMed, Web of Science, Embase and Cochrane Library until March 2025. Published studies reporting adjusted odds ratios (ORs), hazard ratios (HRs), or relative risks (RRs) for stroke in perceived psychosocial stress versus non-perceived psychosocial stress individuals and perceived stress in stroke versus non-stroke individuals were included. A random-effects model was used to pool effect estimates, with heterogeneity assessed via the chi-square test based on Cochrane Q statistics. Subgroup evaluations were conducted for stroke type (ischemic/hemorrhagic), region, and sex.

**Results:**

Eleven case–control studies were included, with the case group comprising stroke patients (*n* = 21,024) and the control group consisting of healthy individuals matched for other characteristics (*n* = 22,408). Meta-analysis revealed a higher incidence of perceived psychosocial stress in the stroke population compared to the control group (RR = 1.58, 95% CI [1.17–1.80], *p* < 0.00001, *I*^2^ = 84%), with statistically significant differences. Subgroup analyses were conducted for regions: Europe and America (RR = 1.53, 95% CI [1.19–1.95], *p* = 0.0007); Asia (RR = 2.10, 95% CI [1.37–2.91], *p* = 0.0006); stroke types: ischemic stroke (RR = 1.58, 95% CI [1.30–1.91], *p* < 0.0001); hemorrhagic stroke (RR = 1.43, 95% CI [1.33–1.53], *p* < 0.00001); Sex: Male (RR = 1.58, 95% CI [1.44–1.73], *p* < 0.00001); Female (RR = 1.38, 95% CI [1.27–1.51], *p* < 0.00001); and Age < 50 years (RR = 1.50, 95% CI [1.24–1.83], *p* < 0.0001). Fourteen prospective cohort studies were included, with 107,741 participants in the perceived stress group and 69,784 in the control group. The results showed that the perceived stress group had a higher probability of stroke than the control group, but the difference was not statistically significant (RR = 1.29, 95% CI [0.83–2.02], *p* = 0.26, *I*^2^ = 99%). Subgroup analyses for Europe and America (RR = 1.65, 95% CI [0.88–3.07], *p* = 0.12) and Asia (RR = 1.06, 95% CI [0.82–1.38], *p* = 0.64) also showed no statistically significant differences. No significant associations were found for ischemic stroke (RR = 0.94, 95% CI [0.75–1.17], *p* = 0.57), hemorrhagic stroke (RR = 0.97, 95% CI [0.73–1.29], *p* = 0.83), or by Sex (Male: RR = 1.95, 95% CI [0.87–4.36], *p* = 0.11; Female: RR = 0.59, 95% CI [0.18–1.96], *p* = 0.39).

**Conclusion:**

Meta-analysis of case–control studies demonstrated that perceived psychosocial stress is a risk factor for stroke, ischemic stroke, and hemorrhagic stroke in both Europe/America and Asia, regardless of sex, particularly among stroke patients Age < 50. However, prospective cohort studies revealed no significant differences in the probability of stroke, ischemic stroke, or hemorrhagic stroke between the perceived stress and control groups across regions (Europe/America and Asia) and Sex (males and females).

**Meta-analysis registration:**

PROSPERO2025; https://www.crd.york.ac.uk/PROSPERO/view/CRD420251012354.

## 1 Introduction

Stroke affects up to one in five people during their lifetime in some high-income countries, and up to almost one in two in low-income countries. Globally, it is the second leading cause of death, stroke includes ischemic stroke and hemorrhagic stroke, ischemic strokes constitute between 60 and 70% of all strokes and result from an acute arterial occlusion (1). Psychological or psychosocial stress occurs when an individual perceives that environmental demands tax or exceed his or her adaptive capacity. Studies of psychological stress focus either on the occurrence of environmental events that are consensually judged as taxing one’s ability to cope or on individual responses to events that are indicative of this overload, such as perceived stress and event-elicited negative affect. Widespread public belief that psychological stress leads to disease, as clinical depression, cardiovascular disease, human immunodeficiency virus (HIV)/AIDS, and cancer (2). Although the increasing prevalence of stroke and psychological stress has led to numerous clinical studies reporting correlations between perceived stress and stroke, whether psychological stress constitutes a high-risk factor for stroke remains inconclusive. Therefore, a meta-analysis was conducted to evaluate the association between psychological stress and stroke.

## 2 Methods

### 2.1 Search strategy

The meta-analysis was conducted in strict accordance with the PRISMA (Preferred Reporting Items for Systematic Reviews and Meta-Analyses) guidelines (3). The study protocol has been registered in the International Prospective Register of Systematic Reviews (PROSPERO - registration number: CRD420251012354). Based on the PRISMA guidelines, we performed a systematic search of online databases (PubMed, Web of Science, Embase and the Cochrane Library) before March 2025. The following search terms were used in our search strategies: (‘stroke’ or ‘brain ischemic’ or ‘transient brain ischemia’ or ‘cerebra arterial disease’ or ‘ischemic stroke’ or ‘non-ischemic stroke’ or ‘Hemorrhage stroke’ or ‘cerebrovascular accident’ or ‘cerebrovascular disorders’ or ‘TIA’ or ‘intracerebral hemorrhage’ or ‘brain Infarction’) AND (‘psychological stress’ or ‘psychosocial stress ‘or ‘psychological distress’ or ‘stress’).

### 2.2 Inclusion criteria

The identified studies were included for the meta-analysis if they fulfilled the following criteria: (1) Only peer-reviewed published literature, including observational studies (cohort studies and case–control studies) and randomized controlled trials (RCTs); (2) Based on human subjects; (3) Exposure factors were perceived stress, perceived psychosocial stress, or stroke, with clearly defined diagnostic criteria for stroke and perceived psychological/social stress; (4) Each study must include either a stroke patient group with a non-stroke control group, or a perceived psychosocial stress group with a non-perceived psychosocial stress control group, and provide effect estimates (relative risk [RR], odds ratio [OR], or hazard ratio [HR]) with corresponding 95% confidence intervals (CI) for the association between perceived stress and stroke risk, or sufficient raw data to calculate these estimates.

### 2.3 Exclusion criteria

In the process of literature screening, the following items of research were excluded: (1) case reports, conference abstracts, review papers, editorials, commentaries; (2) non-English literature; (3) articles without sufficient data to assess the association between psychological stress and stroke risk.

### 2.4 Data abstraction and quality assessment

All data were independently extracted by two reviewers using a standardized data collection table. Discrepancies in data extraction were resolved by consensus. We extracted the following data from each study: first author’s name, publication year, region, Grade the degree of stress, length of follow-up, Sex, Stroke subtype measurement, the quality of each study was assessed by the Newcastle–Ottawa Scale (NOS), a standard commonly used to assess quality in cohort studies (4). The scoring system consisted of three parts: population selection, comparability between groups, and exposure factors. Results ranged from 0 to 9, with higher scores indicating better quality of the method (Table 1).

**Table 1:** Characteristics of included case-control studies and prospective cohort studies.

	Case–control studies						
	First author (year)	Region	Previous stroke excluded	Grade the degree of stress	Stroke subtype measurement	Sex	NOS score
1	Holm, 2025 (5)	Sweden	Yes	No	No	–	9
2	Kutal, 2025 (6)	19 centers in European	Yes	Yes	No	M/F	7
3	Maalouf, 2023 (14)	Lebanon	Yes	No	Yes (ischemic)	–	9
4	Reddin, 2022 (7)	Asia, USA… (32 countries)	Yes	Yes	Yes (both)	–	9
5	Prasad, 2020 (15)	India	Unknown	No	No	M/F	7
6	Sarfo, 2018 (13)	15 centers in Nigeria and Ghana	Yes	No	No	–	9
7	Wilde, 2018 (8)	Netherlands	Yes	Yes	YES (hemorrhagic)	–	9
8	Egido, 2012 (9)	Spain	Yes	Yes	No	–	8
9	O’Donne, 2010 (12)	84 centers in Argentina, Australia… (22 countries)	Yes	No	Yes (both)	–	9
10	Jood, 2009 (10)	Sweden	Unknown	Yes	Yes (ischemic)	–	9
11	Abel, 1999 (11)	USA	Yes	Yes	Yes (ischemic)	–	9

	Prospective cohort studies						
	First author (year)	Region	Follow-up years (y)	Grade the degree of stress	Stroke subtype measurement	Sex	NOS score
1	Li, 2022 (16)	China	4.5	Yes	Yes (both)	–	8
2	Hagström, 2018 (17)	Sweden, USA… (39 countries)	3.7	No	No	–	8
3	Susan, 2014 (18)	USA	8.5	Yes	Yes (ischemic)	M/F	9
4	Molshatzki, 2013 (19)	Israel	28.1	Yes	No	–	8
5	Henderson, 2013 (20)	USA	6	Yes	No	–	8
6	Suadicani, 2011 (21)	Denmark	30	No	No	M	7
7	Kornerup, 2010 (22)	Denmark	6–9	Yes	Yes (ischemic)	–	9
8	Tsutsumi, 2009 (23)	Japan	11	Yes	Yes (both)	M/F	9
9	Harmsen, 2006 (24)	Sweden	28	No	No	M	8
10	Ohlin, 2004 (25)	Sweden	21	Yes	No	M/F	8
11	Truelsen, 2003 (26)	Denmark	14–16	Yes	No	–	9
12	Iso, 2002 (27)	Japan	7.9	Yes	Yes (both)	M/F	9
13	Macleod, 2001 (28)	UK	21	Yes	No	–	8
14	Susan, 2001 (50)	Finland	11	Yes	Yes (both)	M	7

NOS, Newcastle–Ottawa Scale; M, male; F, female.

### 2.5 Statistical analysis

Results from cohort studies and case–control studies are usually expressed in terms of relative risk (RR). Heterogeneity among studies was assessed using the chi-square test based on Cochrane *Q* statistics at *p* < 0.05 level of significance, and quantification of heterogeneity was made by the I^2^ metric, which describes the estimated percentage of variability for effects due to differences rather than chance. When *I*^2^ > 50%, there was significant statistical heterogeneity in this study. To explore possible explanations for homogeneity and test the robustness of the association between psychological stress and risk of stroke, we conducted sensitivity analyses and subgroup analyses by stroke type, region, sex, questionnaire. To identify possible sources of heterogeneity, a meta-regression analysis was conducted by including covariates such as stroke type, region, sex, questionnaire. Publication bias was evaluated visually by creating funnel plots via Review Manager 5.4 version (Cochrane Collaboration, Oxford, UK),as well as by conducting Egger’s regression test using Stata 18.0 Version (Stata Corp, College Station, TX, USA) for outcomes with 10 or more include studies *p* value < 0.05 was considered as statistically significant publication bias. Cochrane Review The goal of the study was to carry out two separate analyses for the two distinct study designs (i.e., case–control studies and prospective cohort study) identified during our review. A sensitivity analysis was performed, based on excluding one study at a time, to examine the impact of each exclusion on the pooled estimates and variances of the included studies. Manager (Version 5.4) software and Stata 18.0 were used to conduct the meta-analysis and obtain graphical output. *p* values were 2-sided and *p* < 0.05 was considered statistically significant.

## 3 Results

### 3.1 Study characteristics

**Figure 1:**
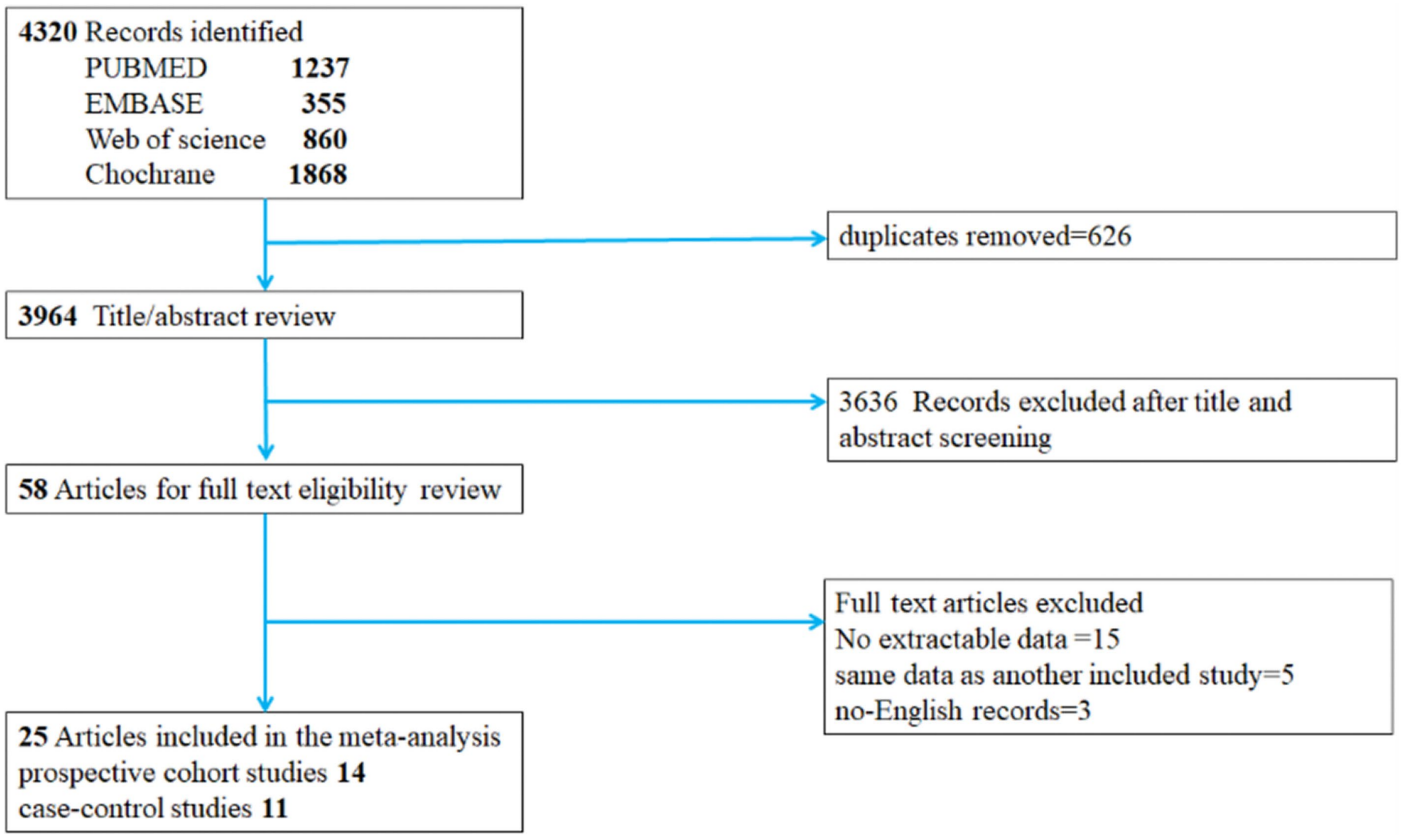
Literature screening flow chart. Prospective cohort studies case–control studies.

The systematic search identified 4,320 articles from online databases that were subsequently examined on title and abstract. Figure 1 shows the stages in obtaining studies for inclusion in the review. Finally, a total of 25 articles with 14 prospective cohort studies and 11 case–control studies were included in the meta-analysis. The Newcastle–Ottawa Scale was uniformly applied to assess methodological quality across all 25 included studies (Table 1).

Eleven case–control studies were included, with distribution as follows: by region—7 studies in Europe/America (5–11), 3 in Asia (8, 9, 12), and 2 in Africa (7, 13); by stroke type—7 ischemic strokes (6, 7, 10–12, 14, 15) and 3 hemorrhagic strokes (7, 8, 12); by Sex—2 Male (6, 7) and 2 Female (6, 7); 9 studies with perceived stress questionnaire scores or severity grade (5–12, 14); and 3 studies focusing on participants aged < 50 years (6, 7, 13) (Table 2).

**Table 2:** Subgroup analysis characteristics of case–control studies.

Author year	Region	Stroke group: control group	No. of perceived stress, stroke group: control group	Questionnaire
1. Holm, 2025 (5)	Sweden	Stroke: control = 50: 100	Stroke: control = 36: 52	Yes
2. Kutal, 2025 (6)	19 European centers were included	Ischemic stroke: control (total) = 426: 426; ischemic stroke: control (Female) = 203: 203; ischemic stroke: control (Male) = 223: 223; age < 40, ischemic stroke: control = 188: 188; age ≥ 40 ischemic stroke: control = 238: 238	At least moderate stress (total) (Q2 + Q3) = 197: 142; high perceived stress (Total) (Q3) = 18: 7; at least moderate stress (female) (Q2 + Q3) = 117: 84; High perceived stress (female) (Q3) = 14: 6. At least moderate stress (male) = 80: 58; high perceived stress (male) = 4: 1. Age < 40, At least moderate stress = 90: 63; high perceived stress = 9: 3. Age ≥ 40岁 At least moderate stress = 107: 79. High perceived stress = 9: 4 Q2 Q3 as stress	Yes
3. Maalouf, 2023 (14)	Lebanon	Ischemic stroke: control = 113: 451	Ischemic stroke: control = 31: 62	Yes
4. Reddin, 2022 (7)	32 countries in Asia, America, Europe, Australia, the Middle East, and Africa	Cases: controls = 13,350: 13,462, Europe + America, cases (stress event): controls (stress event) = 1,240 (902): 1,169 (679); Africa, cases (stress event): controls (stress event) = 351 (265): 337 (248); Asia, cases (stress event): controls (stress event) = 5,551 (1,204): 5,377 (735)	None (Q0) or some periods (Q1) = 10,605: 11,529. Several periods (Q2) or permanent (Q3) = 2,745: 1,933; Q2 Q3 as stress, general stress never (Ischemic, ICH, Control) = 2,772: 760: 4,217; some of the time (Ischemic, ICH, control) = 5,425: 1,615: 7,345; several periods/permanent (Ischemic, ICH, control) = 2,101: 633: 1,944. Age < 45 home stress, never (Ischemic: ICH: control) = 302: 116: 514; some of the time (Ischemic: ICH: control) = 620: 256: 909; several periods/permanent = 198: 75: 155. Men: never (Ischemic: ICH: control) = 2,211: 575: 3,330; some of the time (Ischemic: ICH: control) = 3,175: 1,007: 4,091; several periods/permanent = 750: 222: 615. Woman: never (Ischemic: ICH: control) = 1,236: 388: 1,916; some of the time (Ischemic: ICH: control) = 2,217: 618: 2,842; several periods/permanent = 719: 201: 671.	Yes
5. Prasad, 2020 (15)	India	Ischemic stroke + ICH = 151: 151 (self-control)	Case: control = 38: 9	No
6. Sarfo, 2018 (13)	West Africans;15 sites in Nigeria and Ghana	Age < 50: ischemic stroke + ICH = 515: 515; Age ≥ 50: ischemic stroke + ICH = 1,603:1,603; total = 2,118: 2,118,	Age < 50, case: control = 134: 103; age ≥ 50, case: control = 369: 224; total = 503: 327.	No
7. Wilde, 2018 (8)	Netherlands	UIA: ASAH: controls = 215: 467: 733	Never (Q0): sometimes (Q1): often (Q2): always (Q3) = UIA 78: 79: 42: 16 (215); ASAH:186: 188: 71: 22 (467) control: 323: 307: 87: 16 (733), Q2 Q3 as stress	Yes
8. Egido, 2012 (9)	Spain	Ischemic stroke + ICH = 150: 300	Score > 300 (Q3) Case: control = 16:9; score ≥ 150–300 (Q2) case: control = 46:41; Score < 150 (Q1) = 88: 250 (no never case), Q2 Q3 as stress	Yes
9. O’Donne, 2010 (12)	22 countries	Ischemic stroke: ICH: control = 2,324: 654: 2,987	Ischemic stroke: ICH: control = 465: 124: 440.	Yes
10. Jood, 2009 (10)	Sweden	Ischemic stroke: control = 566: 593	Cases/controls = never experienced stress (Q0) = 56: 51; some period of stress (Q1) = 136: 187; some period of stress during the last 5 years (Q2) = 89: 126; several periods of stress during the last 5 years = 159: 183; permanent stress during the last year (Q3) = 46: 17 permanent stress during the last 5 year (Q4) = 80: 29 Q3 Q4 as stress	Yes
11. Abel, 1999 (11)	USA	Ischemic stroke: control = 655:1087	Case: control = GSRRS score 1–159 (Q1) = 286:464; GSRRS score 160–239 (Q2) = 252:388 GSRRS score 240–399 (Q3) = 86:151 GSRRS score 480–(Q4) = 41:75. Q3 Q4 as stress	Yes

No., number; Q0, questionnaire of perceived Stress never; Q1, questionnaire of perceived stress low or sometimes; Q2, questionnaire of perceived stress moderate or often; Q3, questionnaire of perceived stress high, Q4, questionnaire of perceived stress more than high or very serious; ICH, intracerebral hemorrhage; GSRRS, geriatric social readjustment rating scale.

Fourteen prospective cohort studies were included, among which 13 utilized perceived psychosocial stress questionnaires (16–28). Stratified by region, 7 studies were conducted in Europe and America (20–22, 24–26, 28) and 4 in Asia (16, 19, 23, 27). By stroke type, 4 studies reported ischemic stroke (16, 18, 22, 27) and 2 hemorrhagic stroke (16, 27). By Sex, 5 Males (21, 23–25, 27) and 3 Females (23, 25, 27) (Table 3).

**Table 3:** Characteristics of subgroup analysis in prospective cohort studies.

Author year	Region	Stress group: control	No. of stroke in stress group: control group	PSS or other questionnaire
1. Li, 2022 (16)	China	Psychological stress, high: medium: low = 1,189: 6,921: 12,578.	Stroke 636: high (Q2): medium (Q1): low (Q1) = 46: 197: 393: ischemic stroke, high: medium: low = 7: 164: 313. Hemorrhagic stroke, high: medium: low = 9: 33: 78; (Q2 Q1 as stress)	Yes
2. Hagström, 2018 (17)	39 countries	Psychological stress: rarely/never stress; = 8,694: 6,407	Psychological stress: rarely/never stress = 281: 347	Yes
3. Susan, 2014 (18)	USA	Chronic stress score ≥ 2: chronic stress score 1: chronic stress score0 = 2,324: 2,098: 2,292	Chronic stress score ≥ 2 (Q1): chronic stress score 1 (Q1): chronic stress score 0 (Q0) = 72:64:57. Ischemic stroke, high: low: never = 41: 40: 39 (Q2 Q1 as stress)	Yes
4. Molshatzki, 2013 (19)	Israel	Hardship score: bottom (0–1), middle (2–4) and top (5–14):top: middle: bottom = 2,667: 4,421: 2,941	Stroke, top (Q2): middle (Q1): bottom (Q0) = 194: 307: 164 (Q2 Q1 as stress)	Yes
5. Henderson, 2013 (20)	USA	Quartiles of distress, 总计4,120; Q4: Q3: Q2: Q1 = 1,029: 1,031: 1,028: 1,032. Q1 = never; Q2 34 = stress	Stroke, Q4: Q3: Q2: Q1 = 210: 128: 117: 83.	Yes
6. Suadicani, 2011 (21)	Denmark	Stress: control (rarely) = 1,069: 3,862 (all man)	Stroke, stress: control (rarely) = 309: 470	Yes
7. Kornerup, 2010 (22)	Denmark	Major life events (MLE), MLE > 4: MLE3-4: MLE1-2: MLE0 = 1,830: 1,403: 1,643: 1,643	Ischemic stroke, MLE > 4 (Q3): MLE3-4 (Q2): MLE1-2 (Q1): MLE0 (Q0) = 97: 94: 79: 80 (Q3 Q2 as stress)	Yes
8. Tsutsumi, 2009 (23)	Japan	Total, high: low = 1,638: 1,175. males, high: low = 813: 499; females, high: low = 825: 676	Stroke, total, high: low = 43:18, males, high: low = 28:7. females, high: low = 15: 11	Yes
9. Harmsen, 2006 (24)	Sweden	Stress: control = 1,094: 5,737 (all man)	Stroke, stress: control = 540: 479	Yes
10. Ohlin, 2004 (25)	Sweden	Males, high: low: never = 1,583: 549: 8,489. Females, high: low: never = 353: 188: 2,118, total, high: low: never = 1,936: 737: 10,607.	Stroke, males, high (Q2): low (Q1): never (Q0) = 115: 30: 438. Females, high: low: never = 16: 3: 41; total, high: low: never = 131: 33: 479 (Q21 as stress)	Yes
11. Truelsen, 2003 (26)	Denmark	Stress intensity: high: moderate: light: none = 716: 2,381: 4,521: 4,931	Stroke, high (Q3): moderate (Q2): light (Q1): none (Q0) = 59: 173: 288: 408 (Q3 Q2 as stress)	Yes
12. Iso, 2002 (27)	Japan	Perceived mental stress, male, high: medium: low = 6,891: 18,231: 5,058, female, high: medium: low = 8,656: 27,100: 7,488	Male, total stroke, high (Q3): medium (Q2): low (Q1) = 60:198:83, male, hemorrhagic stroke, high: medium: low = 22: 55: 18. Male, ischemic stroke, high: medium: low = 23: 74: 32; female, total, high: medium: low = 67: 196:5 3. Hemorrhagic stroke, high: medium: low = 16: 44: 6, ischemic stroke, high: medium: low = 21: 59: 19 (Q3 Q2 as stress)	Yes
13. Macleod, 2001 (28)	UK	Perceived stress, high: medium: low = 711: 2,912: 1,765	Stroke; high (Q3): medium (Q2): low (Q1) = 16: 58: 48 (Q3 Q2 as stress)	Yes
14. Susan, 2001 (50)	Finland	SBP > 19 mm Hg (high stress): SBP ≤ 19 mm Hg (low stress) = 2,268: 35	High: low = 112: 1	No

No., number; Q0, questionnaire of perceived stress never; Q1, questionnaire of perceived stress low or sometimes; Q2, questionnaire of perceived stress moderate or often; Q3, questionnaire of perceived stress high; Q4, questionnaire of perceived stress more than high or very serious.

Eleven case–control studies and fourteen prospective cohort studies were included. Based on Questionnaire (Q) assessments or graded classifications of perceived psychosocial stress from the incorporated literature, the following criteria were applied for defining perceived stress groups: 1. Perceived stress (Q1): no perceived stress (Q0), with Q1 included in the perceived stress group. 2. High perceived stress (Q3): moderate perceived stress (Q2): low perceived stress (Q1), the perceived stress group (Q3 + Q2) versus the control group (Q1), with Q3 and Q2 included in the perceived stress group. 3. High perceived stress (Q3): moderate perceived stress (Q2): low perceived stress (Q1): no (or almost no) perceived stress (Q0), the perceived stress group (Q3 + Q2) versus the control group (Q0), with Q3 and Q2 included in the perceived stress group. 4. Extreme perceived stress (Q4): high perceived stress (Q3): moderate perceived stress (Q2): low perceived stress (Q1), the perceived stress group (Q4 + Q3) versus the control group (Q1), with Q4 and Q3 included in the perceived stress group.

### 3.2 Eleven case–control studies were included, comparing the occurrence of perceived psychosocial stress between stroke patients and healthy controls, to determine the association between perceived stress and stroke

#### 3.2.1 Comparison of perceived stress between stroke populations and healthy controls

**Figure 2:**
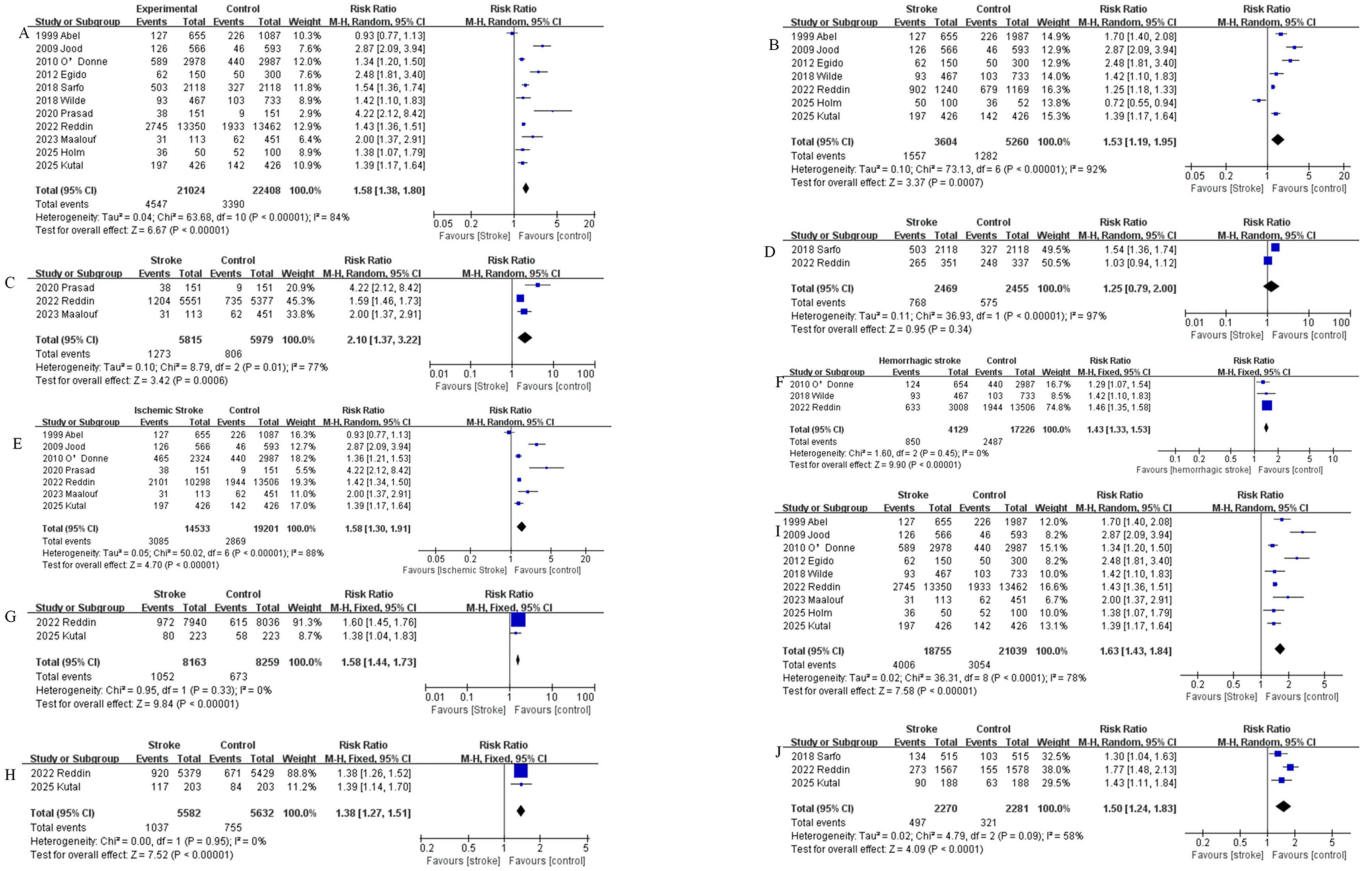
**(A)** Comparison of perceived stress incidence between stroke patients and healthy controls. **(B)** Comparison of perceived stress incidence between stroke patients and healthy controls in studies conducted in Europe and the America. **(C)** Comparison of perceived stress incidence between stroke patients and healthy controls in studies conducted in Asia. **(D)** Comparison of perceived stress between stroke populations and healthy controls in Africa regions. **(E)** Comparison of perceived stress between Ischemic stroke populations and healthy controls. **(F)** Comparison of perceived stress between hemorrhagic stroke populations and healthy controls. **(G)** Male comparison of perceived stress between stroke populations and healthy controls. **(H)** Female comparison of perceived stress between stroke populations and healthy controls. **(I)** Comparison of perceived stress between stroke populations using questionnaires and healthy controls. **(J)** Comparison of perceived stress between Age <50 stroke populations and healthy controls.

Eleven case–control studies were included, with an intervention group of stroke patients (*n* = 21,024) and a control group (*n* = 22,408) of healthy individuals matched to the intervention group for other characteristics. Significant heterogeneity was observed among studies (*I*^2^ = 84%). A random-effects model was employed for meta-analysis, revealing a statistically significant higher incidence of perceived stress in the stroke group compared to controls (RR = 1.58, 95% CI [1.17–1.80], *p* < 0.00001). See Figure 2A.

#### 3.2.2 Comparison of differences in perceived stress incidence between stroke patients and healthy controls across different regions

Comparison of perceived stress between stroke populations and healthy controls in European and American regions. Seven case–control studies conducted in Europe and the Americas were included, comprising 3,604 stroke patients and 5,260 control subjects. Significant heterogeneity was observed among studies (*I*^2^ = 92%). A random-effects model was employed for meta-analysis, revealing a statistically significant higher incidence of perceived stress in the stroke group compared to controls (RR = 1.53, 95% CI [1.19–1.95], *p* = 0.0007). See Figure 2B.

**Figure 3:**
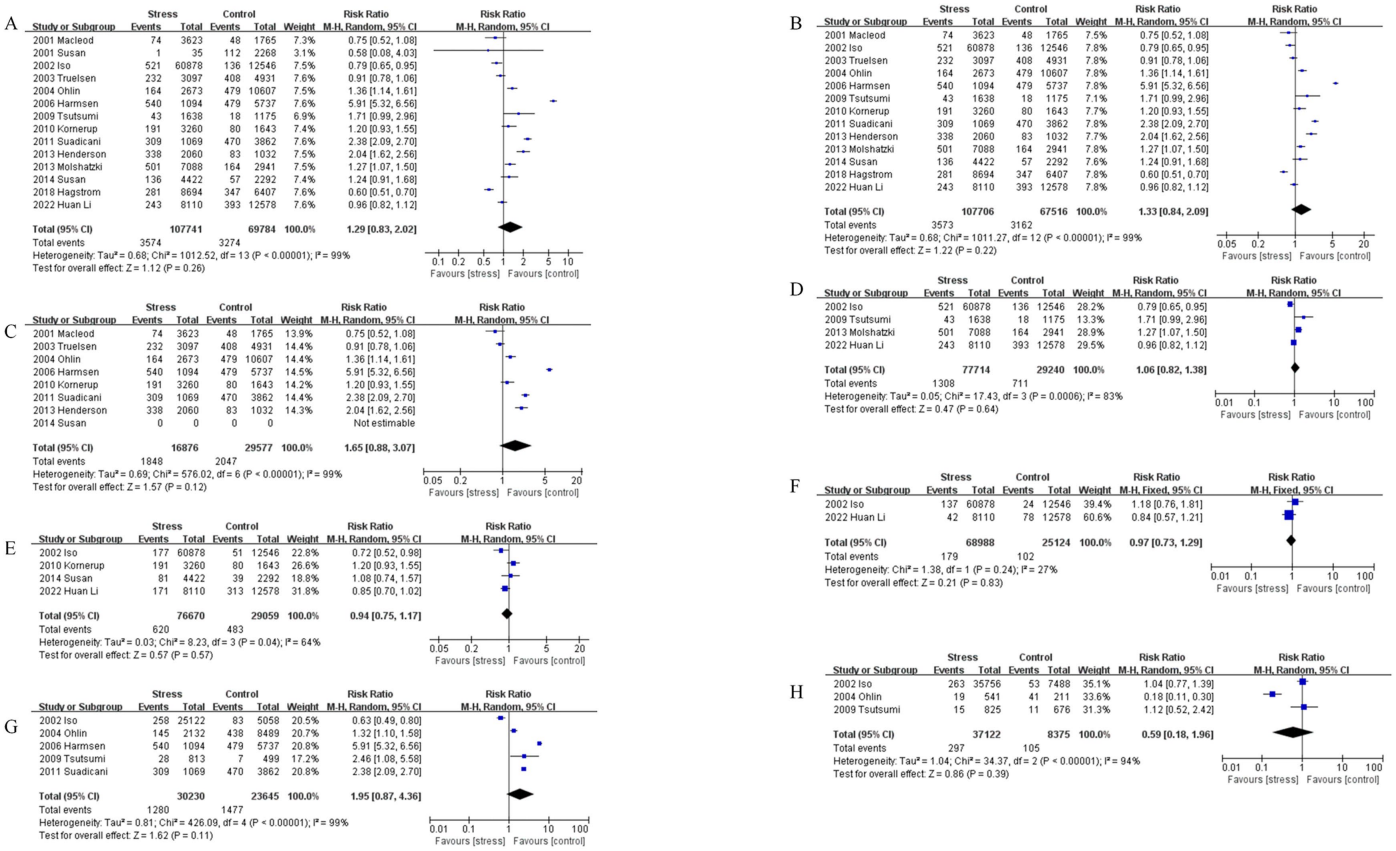
**(A)** Comparison of stroke incidence between populations with perceived stress and control groups. **(B)** Comparison of stroke incidence between populations with perceived stress using questionnaires and control groups. **(C)** Comparison of stroke incidence between populations with perceived stress and control groups in European and American regions. **(D)** Comparison of stroke incidence between populations with perceived stress and control groups in Asia regions. **(E)** Comparison of ischemic stroke risk between populations with perceived stress and control groups. **(F)** Comparison of hemorrhagic stroke risk between populations with perceived stress and control groups.

Comparison of perceived stress between stroke populations and healthy controls in Asia regions. Three case–control studies conducted in Asia were included, comprising 5,815 stroke patients and 5,979 control subjects. Significant heterogeneity was observed among studies (*I*^2^ = 77%). A random-effects model was employed for meta-analysis, demonstrating a statistically significant higher incidence of perceived stress in the stroke group compared to controls (RR = 2.10, 95% CI [1.37–2.91], *Z* = 3.42, *p* = 0.0006). See Figure 3C.

Comparison of perceived stress between stroke populations and healthy controls in Africa regions. Two case–control studies conducted in Africa were included, comprising 2,469 stroke patients and 2,455 control subjects. There was heterogeneity among the studies (*I*^2^ = 97%). A random-effects model was employed for meta-analysis. The results showed that the incidence of perceived stress was higher in the stroke group than in the control group, but the difference was not statistically significant (RR = 1.25, 95% CI [0.79–2.00], *Z* = 0.95, *p* = 0.34). See Figure 2D.

#### 3.2.3 Comparison of differences in perceived stress incidence among ischemic stroke patients, hemorrhagic stroke patients and healthy controls

Seven case–control studies on ischemic stroke were included, comprising 14,533 ischemic stroke patients and 19,201 control subjects. Significant heterogeneity was observed among studies (*I*^2^ = 88%). A random-effects model was employed for meta-analysis, revealing a statistically significant higher incidence of perceived stress in the ischemic stroke group compared to controls (RR = 1.58, 95% CI [1.30–1.91], *p* < 0.0001). See Figure 2E. Three case–control studies on hemorrhagic stroke were included, comprising 4,129 hemorrhagic stroke patients and 17,226 control subjects. No significant heterogeneity was observed among studies (*I*^2^ = 0%). A fixed-effects model was employed for meta-analysis, demonstrating a statistically significant higher incidence of perceived stress in the hemorrhagic stroke group compared to controls (RR = 1.43, 95% CI [1.33–1.53], *p* < 0.00001). See Figure 2F.

#### 3.2.4 Comparison of perceived stress incidence between stroke patients and healthy controls by sex

Two case–control studies in males were included, comprising 8,163 stroke patients and 8,259 healthy controls. No significant heterogeneity was observed among studies (*I*^2^ = 0%). A fixed-effects model was employed for meta-analysis, demonstrating a statistically significant higher incidence of perceived stress in the stroke group compared to controls (RR = 1.58, 95% CI [1.44–1.73], *p* < 0.00001). See Figure 2G. Two case–control studies in females were included, comprising 5,582 stroke patients and 5,632 healthy controls. No significant heterogeneity was observed among studies (*I*^2^ = 0%). A fixed-effects model was employed for meta-analysis, demonstrating a statistically significant higher incidence of perceived stress in the stroke group compared to controls (RR = 1.38, 95% CI [1.27–1.51], *p* < 0.00001). See Figure 2H.

#### 3.2.5 Comparison of perceived stress between stroke populations using questionnaires and healthy controls

Case–control studies utilized detailed perceived stress questionnaires, such as the PSS or other questionnaires. Nine case–control studies were included, comprising 18,755 stroke patients and 21,039 control subjects. Significant heterogeneity was observed among studies (*I*^2^ = 78%). A random-effects model was employed for meta-analysis, demonstrating a statistically significant higher incidence of perceived stress in the stroke group compared to controls (RR = 1.63, 95% CI [1.43–1.84], *p* < 0.0001). See Figure 2I.

#### 3.2.6 Comparison of perceived stress incidence between stroke patients aged < 50 years and healthy controls

Three case–control studies of stroke in patients aged < 50 years were included, comprising 2,270 stroke patients and 2,281 control subjects. Significant heterogeneity was observed among studies (*I*^2^ = 58%). A random-effects model was employed for meta-analysis, demonstrating a statistically significant higher incidence of perceived stress in the stroke group compared to controls (RR = 1.50, 95% CI [1.24–1.83], *p* < 0.0001). See Figure 2J.

### 3.3 Fourteen cohort studies were included to compare the risk of stroke between the perceived stress group and the control group, clarifying the correlation between perceived stress and stroke

#### 3.3.1 Comparison of stroke incidence between the perceived stress group and the control group

Fourteen prospective cohort studies were included, comprising 107,741 subjects in the perceived stress group and 69,784 in the control group. Significant heterogeneity was observed among studies (*I*^2^ = 99%). A random-effects model was employed for meta-analysis. The results showed a higher risk of stroke in the perceived stress group compared to the control group, but the difference was not statistically significant (RR = 1.29, 95% CI [0.83–2.02], *p* = 0.26). See Figure 3A.

#### 3.3.2 Comparison of stroke incidence between the perceived stress group with questionnaires and the control group

Thirteen prospective cohort studies with questionnaires were included, comprising 107,706 subjects in the perceived stress group and 67,516 in the control group. Significant heterogeneity was observed among studies (*I*^2^ = 99%). A random-effects model was employed for meta-analysis. The results showed a higher risk of stroke in the perceived stress group compared to the control group, but the difference was not statistically significant (RR = 1.33, 95% CI [0.84–2.09], *p* = 0.22). See Figure 3B.

#### 3.3.3 Comparison of stroke incidence between the perceived stress group and the control group in different regions

Seven prospective cohort studies in regions of Europe and America were included, comprising 16,876 subjects in the perceived stress group and 29,577 in the control group. Significant heterogeneity was observed among studies (*I*^2^ = 99%). A random-effects model was employed for meta-analysis. The results showed a higher risk of stroke in the perceived stress group compared to the control group, but the difference was not statistically significant (RR = 1.65, 95% CI [0.88–3.07], *p* = 0.12). See Figure 3C. Four prospective cohort studies in regions of Asia were included, comprising 77,714 subjects in the perceived stress group and 29,240 in the control group. Significant heterogeneity was observed among studies (*I*^2^ = 83%). A random-effects model was employed for meta-analysis. The results showed a higher risk of stroke in the perceived stress group compared to the control group, but the difference was not statistically significant (RR = 1.06, 95% CI [0.82–1.38], *p* = 0.64). See Figure 3D.

#### 3.3.4 Comparison of stroke incidence between the perceived stress group and the control group for ischemic stroke or hemorrhagic stroke

Four prospective cohort studies on ischemic stroke were included, comprising 76,670 subjects in the perceived stress group and 29,059 in the control group. Significant heterogeneity was observed among studies (*I*^2^ = 64%). A random-effects model was employed for meta-analysis. The results showed a lower risk of ischemic stroke in the perceived stress group compared to the control group, but the difference was not statistically significant (RR = 0.94, 95% CI [0.75–1.17], *p* = 0.57). See Figure 3E. Two prospective cohort studies on hemorrhagic stroke were included, comprising 68,988 subjects in the perceived stress group and 25,124 in the control group. Significant heterogeneity was observed among studies (*I*^2^ = 27%). A fixed-effects model was employed for meta-analysis. The results showed a lower risk of hemorrhagic stroke in the perceived stress group compared to the control group, but the difference was not statistically significant (RR = 0.97, 95% CI [0.73–1.29], *p* = 0.83). See Figure 3F.

#### 3.3.5 Comparison of stroke incidence between the perceived stress group and the control group by sex

Five prospective cohort studies comparing males in the perceived stress group and the control group were included, comprising 30,230 subjects in the perceived stress group and 23,645 in the control group. Significant heterogeneity was observed among studies (*I*^2^ = 99%). A random-effects model was employed for meta-analysis. The results showed a higher risk of stroke in the perceived stress group compared to the control group, but the difference was not statistically significant (RR = 1.95, 95% CI [0.87–4.36], *p* = 0.11). See Figure 3G. Three prospective cohort studies comparing females in the perceived stress group and the control group were included, comprising 37,122 subjects in the perceived stress group and 8,375 in the control group. Significant heterogeneity was observed among studies (*I*^2^ = 94%). A random-effects model was employed for meta-analysis. The results showed a lower risk of stroke in the perceived stress group compared to the control group, but the difference was not statistically significant (RR = 0.59, 95% CI [0.18–1.96], *p* = 0.39). See Figure 3H.

### 3.4 Publication Bias assessment

#### 3.4.1 Comparison of perceived stress incidence between stroke patients and healthy controls

**Figure 4:**
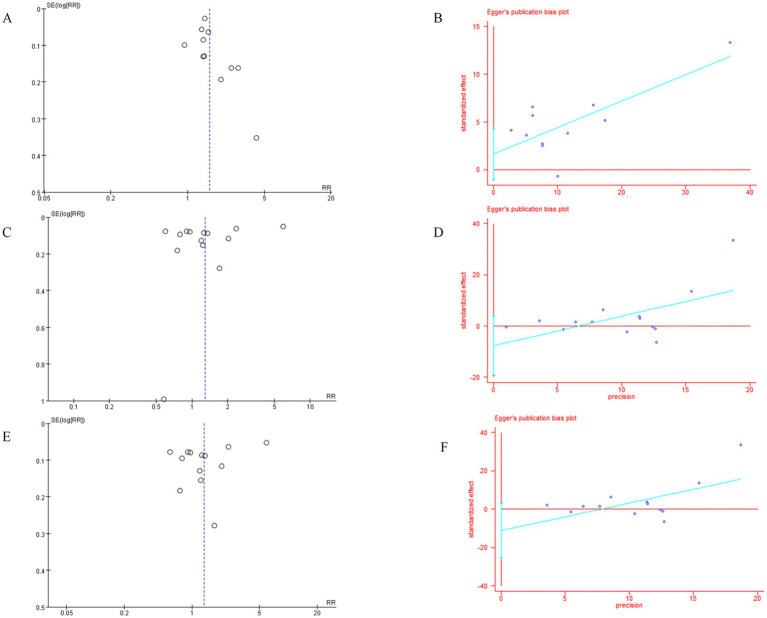
**(A)** Funnel plot for the comparison of perceived stress incidence between stroke patients and healthy controls; **(B)** Egger’s plot for the comparison of perceived stress incidence between stroke patients and healthy controls; **(C)** Funnel plot for the comparison of stroke incidence between the perceived stress group and control group; **(D)** Egger’s plot for the comparison of stroke incidence between the perceived stress group and control group; **(E)** Funnel plot for the comparison of stroke incidence between the perceived stress group with questionnaires and control group; **(F)** Egger’s plot for the comparison of stroke incidence between the perceived stress group with questionnaires and control group.

Funnel plot analysis showed a slightly asymmetric distribution of studies on both sides of the funnel, suggesting possible publication bias (see Figure 4A). Egger’s test (*p* = 0.204) indicated no statistically significant publication bias (see Figure 4B).

#### 3.4.2 Comparison of stroke incidence between the perceived stress group and the control group

Funnel plot analysis showed a symmetric distribution of studies on both sides of the funnel, suggesting no potential publication bias (see Figure 4C). Egger’s test (*p* = 0.167) indicated no statistically significant publication bias (see Figure 4D).

#### 3.4.3 Comparison of stroke incidence between the perceived stress group with questionnaires and the control group

Funnel plot analysis showed a symmetric distribution of studies on both sides of the funnel, suggesting no potential publication bias (see Figure 4E). Egger’s test (*p* = 0.117) indicated no statistically significant publication bias (see Figure 4F).

### 3.5 Sensitivity analysis

**Figure 5:**
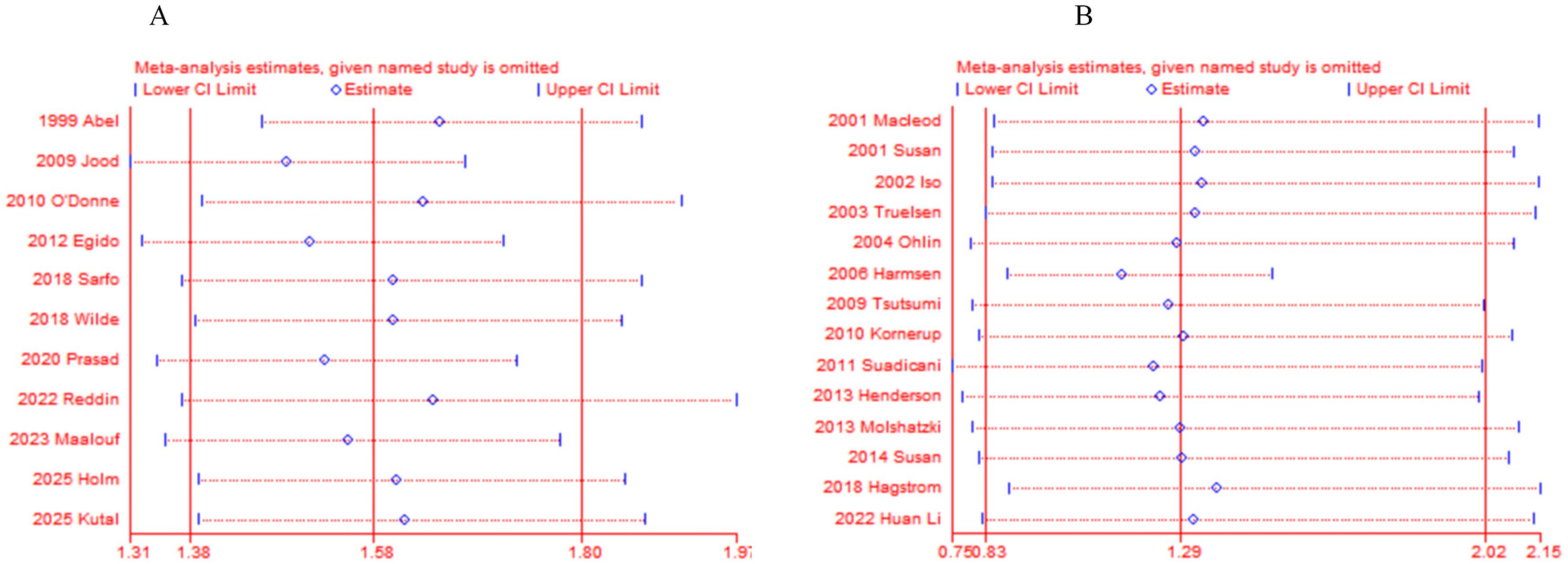
**(A)** Sensitivity analysis of 11 case–control studies. **(B)** Sensitivity analysis of 14 prospective cohort studies.

After performing sensitivity analysis by excluding each study one by one for 11 case–control studies and 14 cohort studies, we found that the results of this meta-analysis were not influenced by any single study. Even if a particular study was removed, the overall conclusions of the study did not change significantly. Therefore, the results of this meta-analysis are stable. See (Tables 4, 5, Figures 5A,B).

**Table 4:** Sensitivity analysis of perceived stress incidence in 11 case–control studies comparing stroke patients and healthy controls.

Study omitted	OR [95% CI]
Abel, 1999 (11)	1.6195067 [1.4114066–1.8582894]
Jood, 2009 (10)	1.5167219 [1.3731627–1.6752897]
O’Donne, 2010 (12)	1.6990612 [1.4553246–1.9836186]
Egido, 2012 (9)	1.5505491 [1.3820884–1.7395432]
Wilde, 2018 (8)	1.657202 [1.4440841–1.901772]
Reddin, 2022 (7)	1.6971816 [1.425347–2.0208592]
Maalouf, 2023 (14)	1.6008395 [1.4072037–1.8211203]
Holm, 2025 (5)	1.6612555 [1.447961–1.9059697]
Kutal, 2025 (6)	1.6757952 [1.4505315–1.9360418]
Combined	1.6257582 [1.4337913–1.843427]

**Table 5:** Sensitivity analysis of 14 prospective cohort studies.

Study omitted	OR [95% CI]
Macleod, 2001 (28)	1.3474317 [0.84631722–2.1452621]
Susan, 2001 (50)	1.325391 [0.84158668–2.0873206]
Iso, 2002 (27)	1.3438475 [0.84120717–2.1468269]
Truelsen, 2003 (26)	1.3282992 [0.82455037–2.1398073]
Ohlin, 2004 (25)	1.284174 [0.78963905–2.088426]
Harmsen, 2006 (24)	1.1506409 [0.8772488–1.5092348]
Tsutsumi, 2009 (23)	1.2646249 [0.79392336–2.0143962]
Kornerup, 2010 (22)	1.2979744 [0.80834561–2.0841799]
Suadicani, 2011 (21)	1.2261654 [0.74734759–2.0117569]
Henderson, 2013 (20)	1.2435148 [0.77147068–2.0043913]
Molshatzki, 2013 (19)	1.2915322 [0.79494911–2.0983172]
Susan, 2014 (18)	1.2953059 [0.80888487–2.0742351]
Hagstrom, 2018 (17)	1.3779106 [0.88306787–2.1500472]
Li, 2022 (16)	1.3218418 [0.81866891–2.1342764]
Combined	1.2914572 [0.82602931–2.0191313]

### 3.6 GRADE evidence quality assessment

The GRADE approach (29) was used to evaluate the evidence quality for each outcome. Assessments were conducted across five domains: Limitations, inconsistency, indirectness, imprecision, and publication bias. GRADE evidence grading indicated that the evidence levels for primary outcomes were moderate or high quality, as shown in Table 6.

**Table 6:** Evidence classification of GRADE.

Outcome indicators	No. of studies	Quality	Assessment					
		Limitations^a^	Inconsistency	Indirectness	Imprecision	Publication bias	Relative risk (95% CI)	Quality of evidence^b^
1. Perceived stress incidence	11	Serious limitations	No serious inconsistency	No serious indirectness	No serious imprecision	Undetected	1.58 [1.17–1.80]	㊉㊉㊉◯
2. European and American regions	7	Serious limitations	No serious inconsistency	No serious indirectness	No serious imprecision	Undetected	1.53 [1.19–1.95]	㊉㊉㊉◯
3. Asia regions	3	Serious limitations	No serious inconsistency	No serious indirectness	No serious imprecision	Undetected	2.10 [1.37–2.91]	㊉㊉㊉◯
4. Africa regions	2	Serious limitations	No serious inconsistency	No serious indirectness	No serious imprecision	Undetected	1.25 [0.79–2.00]	㊉㊉㊉㊀
5. Ischemic stroke	7	Serious limitations	No serious inconsistency	No serious indirectness	No serious imprecision	Undetected	1.58 [1.30–1.91]	㊉㊉㊉◯
6. Hemorrhagic stroke	3	Serious limitations	No serious inconsistency	No serious indirectness	No serious imprecision	Undetected	1.43 [1.33–1.53]	㊉㊉㊉◯
7. Male	2	Serious limitations	No serious inconsistency	No serious indirectness	No serious imprecision	Undetected	1.58 [1.44–1.73]	㊉㊉㊉◯
8. Female	2	Serious limitations	No serious inconsistency	No serious indirectness	No serious imprecision	Undetected	1.38 [1.27–1.51]	㊉㊉㊉◯
9. Questionnaires	9	Serious limitations	No serious inconsistency	No serious indirectness	No serious imprecision	Undetected	1.63 [1.43–1.84]	㊉㊉㊉◯
10. Age < 50	3	Serious limitations	No serious inconsistency	No serious indirectness	No serious imprecision	Undetected	1.50 [1.24–1.83]	㊉㊉㊉◯
11.Stroke incidence	14	No serious limitations	No serious inconsistency	No serious indirectness	No serious imprecision	Undetected	1.29 [0.83–2.02]	㊉㊉㊉㊉
12. Questionnaires	13	No serious limitations	No serious inconsistency	No serious indirectness	No serious imprecision	Undetected	1.33 [0.84–2.09]	㊉㊉㊉㊉
13. European and American	7	No serious limitations	No serious inconsistency	No serious indirectness	No serious imprecision	Undetected	1.65 [0.88–3.07]	㊉㊉㊉㊉
14. Asia regions	4	No serious limitations	No serious inconsistency	No serious indirectness	No serious imprecision	Undetected	1.06 [0.82–1.38]	㊉㊉㊉㊉
15. Ischemic stroke risk	4	No serious limitations	No serious inconsistency	No serious indirectness	No serious imprecision	Undetected	0.94 [0.75–1.17]	㊉㊉㊉㊉
16. Hemorrhagic stroke risk	2	No serious limitations	No serious inconsistency	No serious indirectness	No serious imprecision	Undetected	0.97 [0.73–1.29]	㊉㊉㊉㊉
17. Male	5	No serious limitations	No serious inconsistency	No serious indirectness	No serious imprecision	Undetected	1.95 [0.87–4.36]	㊉㊉㊉㊉
18. Female	3	No serious limitations	No serious inconsistency	No serious indirectness	No serious imprecision	Undetected	0.59 [0.18–1.96]	㊉㊉㊉㊉

Outcome indicators 1–10: case–control studies, Outcome indicators 11–18: prospective cohort studies. GRADE, Grading of Recommendations Assessment, Development, and Evaluation.

^a^Case–control study lack of blinding, ^b^㊉㊉㊉㊉ (High quality): There is a strong belief that the true effect lies close to estimated effect; ㊉㊉㊉◯ (moderate quality): There is a reasonable degree of certainty that the actual effect is near the estimated effect, while there exists a chance of significant difference; ㊉㊉◯◯ (low quality): the confidence in the estimated effect is restricted and there is a possibility of substantial disparity between the actual and estimated effects; ㊉◯◯◯ (very low quality): there is a minimal confidence in the estimated effect and the actual effect is probable to vary from the estimated one considerably.

1. Comparison of perceived stress between stroke populations and healthy controls.

2. Comparison of perceived stress between stroke populations and healthy controls in European and American regions.

3. Comparison of perceived stress between stroke populations and healthy controls in Asia regions.

4. Comparison of perceived stress between stroke populations and healthy controls in Africa regions.

5. Comparison of perceived stress between Ischemic stroke populations and healthy controls.

6. Comparison of perceived stress between hemorrhagic stroke populations and healthy controls.

7. Comparison of perceived stress between male stroke populations and healthy controls.

8. Comparison of perceived stress between female stroke populations and healthy controls.

9. Comparison of perceived stress between stroke populations using questionnaires and healthy controls.

10. Comparison of perceived stress between Age < 50 stroke populations and healthy controls.

11. Comparison of stroke incidence between populations with perceived stress and control groups.

12. Comparison of stroke incidence between populations with perceived stress using questionnaires and control groups.

13. Comparison of stroke incidence between populations with perceived stress and control groups in European and American regions.

14. Comparison of stroke incidence between populations with perceived stress and control groups in Asia regions.

15. Comparison of ischemic stroke risk between populations with perceived stress and control groups.

16. Comparison of hemorrhagic stroke risk between populations with perceived stress and control groups.

17. Comparison of stroke risk between male populations with perceived stress and control groups.

18. Comparison of stroke risk between female populations with perceived stress and control groups.

## 4 Discussion

Stroke is a major global public health issue and one of the leading causes of disability and mortality (30). For stroke prevention, the identification of at-risk individuals and accurate prediction of stroke risk are crucial for interventional preventive measures. Traditional risk assessments incorporate established risk factors such as hypertension, diabetes mellitus, and smoking (31). The role of perceived psychological stress as a potential risk factor for stroke has garnered increasing attention in recent years (32).

This study included 11 case–control studies and 14 prospective cohort studies for a meta-analysis of the association between perceived psychological stress and stroke. The case–control studies primarily compared the difference in perceived psychological stress incidence between stroke patients and healthy controls, while the prospective cohort studies compared the probability of stroke occurrence between the perceived psychological stress group and the control group. Therefore, the data from case–control studies and prospective cohort studies were pooled and analyzed separately in the meta-analysis. The meta-analysis of 11 case–control studies found that the incidence of perceived psychological stress was higher in the stroke group than in the control group (RR = 1.58, 95% CI [1.17–1.80], *p* < 0.00001), with a statistically significant difference. Significant heterogeneity was observed among the studies (*I*^2^ = 84%). A pooled analysis of 9 case–control studies that used questionnaires for perceived psychological stress assessment also showed significant heterogeneity (*I*^2^ = 78%). The incidence of perceived stress was higher in the stroke group compared to the control group, with a statistically significant difference (RR = 1.63, 95% CI [1.43–1.84], *p* < 0.0001). It can be observed that heterogeneity decreased in studies with specific perceived stress questionnaires. However, since nearly every case–control study applied different questionnaires, further analysis based on a single questionnaire for assessing the degree of perceived psychological stress was not feasible. The main sources of heterogeneity were attributed to differences in questionnaires or lack of standardization in stress assessment. Subgroup analysis by region revealed that the incidence of perceived stress was higher in the stroke group than in the control group in Europe and the Americas, with a statistically significant difference (RR = 1.53, 95% CI [1.19–1.95], *p* = 0.0007). In Asia, the incidence of perceived stress was higher in the stroke group than in the control group, with a statistically significant difference (RR = 2.10, 95% CI [1.37–2.91], *p* = 0.0006), and the proportion of perceived psychological stress in Asian stroke patients was higher compared to those in Europe and the Americas (RR 2.1 vs. RR 1.53). However, in Africa, there was no statistically significant difference in perceived stress between the stroke group and the control group (RR = 1.25, 95% CI [0.79–2.00], *p* = 0.34). Subgroup analysis by stroke type showed that the incidence of perceived psychological stress was higher in both ischemic and hemorrhagic stroke patients compared to the control group, with statistically significant differences (RR = 1.58, 95% CI [1.30–1.91], *p* < 0.0001) for ischemic stroke and (RR = 1.43, 95% CI [1.33–1.53], *p* < 0.00001) for hemorrhagic stroke. Subgroup analysis by sex revealed a statistically significant difference in the incidence of perceived stress between stroke patients and healthy controls (Male RR = 1.58, 95% CI [1.44–1.73], *p* < 0.00001), and (Female RR = 1.38, 95% CI [1.27–1.51], *p* < 0.00001), suggesting that male stroke patients have a higher incidence of perceived psychological stress compared to females. Subgroup analysis by age (<50 years) showed that the incidence of perceived stress was higher in the stroke group than in the control group, with a statistically significant difference (RR = 1.50, 95% CI [1.24–1.83], *p* < 0.0001), indicating that perceived psychological stress may be a risk factor for young stroke. The 14 prospective cohort studies included 107,741 subjects in the perceived psychological stress group and 69,784 in the control group. Significant heterogeneity was observed among the studies (*I*^2^ = 99%). The results showed that the probability of stroke occurrence was higher in the perceived stress group than in the control group, but the difference was not statistically significant (RR = 1.29, 95% CI [0.83–2.02], *p* = 0.26). Among the 13 prospective cohort studies that used questionnaires, significant heterogeneity was also observed (*I*^2^ = 99%), and the results still showed a higher probability of stroke in the perceived stress group compared to the control group, but the difference was not statistically significant (RR = 1.33, 95% CI [0.84–2.09], *p* = 0.22). Heterogeneity did not change because there was only one study without a questionnaire. The primary source of heterogeneity was still attributed to differences in questionnaires. Subgroup analysis by region showed no statistically significant difference in the probability of stroke occurrence between the perceived stress group and the control group in Europe and the Americas (RR = 1.65, 95% CI [0.88–3.07], *p* = 0.12) and Asia (RR = 1.06, 95% CI [0.82–1.38], *p* = 0.64). Furthermore, there was no statistically significant difference in the probability of ischemic stroke (RR = 0.94, 95% CI [0.75–1.17], *p* = 0.57) and hemorrhagic stroke (RR = 0.97, 95% CI [0.73–1.29], *p* = 0.83). Similarly, subgroup analysis by sex showed no statistically significant difference in stroke incidence between the perceived stress group and the control group (Male RR = 1.95, 95% CI [0.87–4.36], *p* = 0.11) and (Female RR = 0.59, 95% CI [0.18–1.96], *p* = 0.39). For the 11 case–control studies (Egger’s test *p* = 0.204) and the 14 prospective cohort studies (Egger’s test *p* = 0.167), both showed no publication bias, and sensitivity analysis indicated that the results of the meta-analysis were stable.

The meta-analysis of case–control studies found that perceived psychological stress is a risk factor for stroke, ischemic stroke, and hemorrhagic stroke in both males and females in Europe, the Americas, and Asia, particularly in stroke patients under 50 years of age. However, the prospective cohort studies found no difference in the probability of stroke, ischemic stroke, or hemorrhagic stroke between the perceived psychological stress group and the control group in Europe, the Americas, Asia, males, or females. We consider that the assessment of psychological stress scales in case–control studies may be more accurate, and the results are relatively reliable. In prospective cohort studies, the degree of psychological stress may fluctuate over several years of follow-up, leading to unreliable outcomes in the probability of stroke among individuals with varying levels of perceived psychological stress.

Stress and negative emotions, including depression, anger and hostility, can have adverse effects on health, leading to an increase in the incidence and mortality of cardiovascular diseases (33). Two recent meta-analyses have concluded that people with depression, especially women, have an increased risk of stroke, although most of the evidence comes from homogeneous white people groups (34, 35). Another study shows that a combination of factors such as depressive symptoms, perceived stress, neuroticism and dissatisfaction with life is associated with the incidence of stroke and even the mortality rate of stroke among black people and white people living in the community (20). Anger is a negative emotion associated with hostile personality and aggressive behavior, which is related to the risk of excessive stroke (36) (37). Further research has found that higher levels of depressive symptoms, greater degrees of chronic stress and higher levels of hostility predict an increased risk of stroke and transient ischemic attack (TIA) (18).

The mechanisms underlying the association between psychological stress and stroke are complex and not fully understood, and may be related to the following mechanisms: 1. Long-term exposure to high levels of psychological stress is associated with chronic inflammation, endothelial dysfunction, platelet activation and aggregation, and autonomic nervous system dysfunction (38), and these factors are closely related to atherosclerosis and stroke. 2. Acute psychological stress may trigger qualitative changes in several procoagulant factors such as fibrinogen, factor XII, factor VII, factor VIII, von Willebrand factor, platelet activity, thrombin-antithrombin complexes, fibrin D-dimer, and tissue-type plasminogen activator (39). The resulting procoagulant activity exceeds the fibrinolytic response, thereby promoting a hypercoagulable state and increasing the probability of stroke occurrence; 3. Psychological stress leads to stress-related acute, recurrent short-term increases in blood pressure, vasospasm, and arrhythmia (6); 4. Psychological stress is often associated with behaviors such as smoking, physical inactivity, and poor diet. Unhealthy dietary and lifestyle habits may lead to reduced levels of high-density lipoprotein (HDL) (40). HDL plays a key role in lipid transport, and disruption of this biomarker is associated with an increased risk of stroke. Perceived psychological stress is related to lifestyle behaviors that may negatively affect HDL levels (41), thereby increasing the risk of stroke; 5. Perceived psychological stress may lead to cerebral hypoperfusion, followed by glial cell ischemia, neuronal damage, and an increased risk of stroke (42); 6. Acute psychological stress may activate the sympathetic nervous system, leading to vasoconstriction and plaque rupture in susceptible individuals, thereby triggering stroke events (43); 7. Tawakol et al. (44) further found that amygdala activity, which is involved with experiencing psychological stress, was significantly associated with increased bone marrow activity and arterial inflammation. At the same time, higher perceived psychosocial stress was related to increased intima-media thickness and carotid artery disease by increasing the release of catecholamines and activating sympathetic nerves (32, 45, 46). Stress and negative emotions activate the hypothalamic–pituitary–adrenal axis, leading to changes in glucocorticoids and an increase in circulating catecholamines. They can also affect endothelial dysfunction and platelet activation, and cause abnormalities in neuroendocrine, metabolic and immune functions (47, 48).

Chloe A (49)et al reported that perceived stress is also associated with worse post-stroke functional outcome and greater disability even after accounting for stroke severity, vascular risk factors, and access to acute stroke care. Experimental stroke studies which incorporate social isolation or social defeat stress have shown that both tissue and functional stroke outcome is affected by the increased expression of TNF-*α* and IL-6, increased glucocorticoid production, and suppression of the protooncogene bcl-2. A meta-analysis on perceived stress and stroke by Joanne Booth in 2015 included 14 studies (10 prospective cohort studies and 4 case–control studies) and demonstrated an independent association between psychosocial stress and increased stroke risk (32).

Early identification of high-risk individuals with perceived psychological stress will facilitate the implementation of targeted preventive strategies and interventions to reduce the burden of stroke incidence and mortality.

## 5 Conclusion

The meta-analysis of case–control studies found that perceived psychological stress is a risk factor for stroke, ischemic stroke, and hemorrhagic stroke in both males and females in Europe, America, and Asia, particularly in stroke patients under 50 years of age. However, the prospective cohort studies found no difference in the probability of stroke, ischemic stroke, or hemorrhagic stroke between the perceived psychological stress group and the control group in Europe, America, Asia, males, or females. Perhaps indicating that perceived psychological stress is a risk factor for stroke.

## Data Availability

The datasets presented in this study can be found in online repositories. The names of the repository/repositories and accession number(s) can be found in the article/supplementary material.
